# DNA ligase 1 deficient plants display severe growth defects and delayed repair of both DNA single and double strand breaks

**DOI:** 10.1186/1471-2229-9-79

**Published:** 2009-06-26

**Authors:** Wanda M Waterworth, Jaroslav Kozak, Claire M Provost, Clifford M Bray, Karel J Angelis, Christopher E West

**Affiliations:** 1 CPS, Faculty of Biological Sciences, University of Leeds, Leeds LS2 9JT, UK; 2 Institute of Experimental Botany AS CR, Na Karlovce 1, 160 00 Praha 6, Czech Republic; 3 Faculty of Life Sciences, University of Manchester, Oxford Road, Manchester M13 9PT, UK

## Abstract

**Background:**

DNA ligase enzymes catalyse the joining of adjacent polynucleotides and as such play important roles in DNA replication and repair pathways. Eukaryotes possess multiple DNA ligases with distinct roles in DNA metabolism, with clear differences in the functions of DNA ligase orthologues between animals, yeast and plants. DNA ligase 1, present in all eukaryotes, plays critical roles in both DNA repair and replication and is indispensable for cell viability.

**Results:**

Knockout mutants of *atlig1 *are lethal. Therefore, RNAi lines with reduced levels of AtLIG1 were generated to allow the roles and importance of *Arabidopsis *DNA ligase 1 in DNA metabolism to be elucidated. Viable plants were fertile but displayed a severely stunted and stressed growth phenotype. Cell size was reduced in the silenced lines, whilst flow cytometry analysis revealed an increase of cells in S-phase in *atlig1-RNAi *lines relative to wild type plants. Comet assay analysis of isolated nuclei showed *atlig1*-*RNAi *lines displayed slower repair of single strand breaks (SSBs) and also double strand breaks (DSBs), implicating AtLIG1 in repair of both these lesions.

**Conclusion:**

Reduced levels of *Arabidopsis *DNA ligase 1 in the silenced lines are sufficient to support plant development but result in retarded growth and reduced cell size, which may reflect roles for AtLIG1 in both replication and repair. The finding that DNA ligase 1 plays an important role in DSB repair in addition to its known function in SSB repair, demonstrates the existence of a previously uncharacterised novel pathway, independent of the conserved NHEJ. These results indicate that DNA ligase 1 functions in both DNA replication and in repair of both ss and dsDNA strand breaks in higher plants.

## Background

As sessile, photosynthetic organisms, plants are necessarily exposed to high levels of environmental stresses including UVB, gamma irradiation and heavy metals which increase somatic recombination frequencies in plants and their progeny [1]. In plants, repair of DNA damage products is particularly important because somatic tissues give rise to germ cells at a relatively late stage in development, which means that mutations accumulating in somatic cells from the effects of environmental genotoxins can be passed onto the next generation of plants [2]. Effective cellular response mechanisms have evolved to cope with DNA damage including cell cycle delay or arrest and activation of DNA repair pathways [3].

DNA ligases play essential roles in all organisms by maintaining the physical structure of DNA. These enzymes seal gaps in the sugar-phosphate backbone of DNA that arise during DNA replication, DNA damage and repair. In *Arabidopsis*, as in other eukaryotes, the ligation reaction uses ATP as a cofactor and the involvement of a covalent AMP-ligase intermediate [4]. Eukaryotes have evolved multiple DNA ligase isoforms, with both specific and overlapping roles in the replication and repair of the nuclear and organellar genomes. DNA ligase 1 (LIG1) is present in all eukaryotes where it is required for joining DNA fragments produced during DNA replication. DNA ligase 1 also plays important roles in DNA single strand break (SSB) repair pathways in mammals and yeast. These pathways are less well characterised in plants, but orthologues of several SSB repair genes are identifiable in the genomes of higher plants [5]. *LIG1 *is an essential gene with lethal knockout phenotypes in yeast, mammalian cells and *Arabidopsis *[6-8]. Whilst LIG1 is essential for cell division in yeast and plants, mouse embryos are viable and develop until mid-term without LIG1, indicating that a second ligase may substitute for growth up to this point [9]. Similarly, mouse cell lines deficient in LIG1 are also viable, indicating that other DNA ligase activities can substitute for LIG1 in DNA replication [10]. Interestingly, although plants deficient in AtLIG1 are null, cell division in gametophytes prior to fertilisation appeared unaffected, suggesting that either that a second ligase can partially substitute for DNA ligase 1, or that ligase 1 levels in haploid cells are sufficient to support gametogenesis [8].

DNA ligase 4 (LIG4) is also present in all eukaryotes and mediates the final step in the non-homologous end joining (NHEJ) pathway of DSB repair. However, there are clear differences between eukaryotes regarding the presence of other forms of DNA ligase. Plants lack a DNA ligase III (LIG3) orthologue, which in mammals participates in base excision repair of the nuclear genome and also functions in the maintenance of the mitochondrial genome [11]. Whilst yeast has two DNA ligases (LIG1 and LIG4), there are three DNA ligase genes in *Arabidopsis thaliana*, two of which (LIG1 and LIG4) have been functionally characterised [12]. An additional third DNA ligase unique to plants, termed ligase VI, has been cloned from rice and *Arabidopsis *[13,14] although the *in planta *function of this DNA ligase remains to be determined.

In addition to the nuclear genome plants possess chloroplast and mitochondrial genomes. AtLIG1 has been shown to be targeted to both the nucleus and the mitochondria [15]. This dual targeting is controlled via an evolutionarily conserved posttranscriptional mechanism that involves the use of alternative start codons to translate distinct ligase proteins from a single transcript.

Whilst a role for *Arabidopsis *LIG4 in NHEJ is well established, the role of the other DNA ligases in *Arabidopsis *DNA repair remains unclear. Previous studies have demonstrated that LIG1 is an essential gene in plants, consistent with a non-redundant role in nuclear DNA replication [8]. However, the lethality of AtLIG1 mutations prevents analysis of the potential roles of this enzyme in DNA repair processes in plants. To address this question, we created *Arabidopsis *lines with reduced AtLIG1 levels which were sufficient to allow growth and development, but which produced plants which were potentially compromised in DNA repair. Analysis of these plants identified lines which exhibited growth defects and a reduced capacity for the repair of both SSBs and DSBs, providing evidence that AtLIG1 is involved in recombination pathways in higher plants. This has provided the first report of a role for AtLIG1 in DSB repair and identification of a novel DNA DSB repair pathway in plants.

## Results

### Phenotypic analyses of DNA ligase1 deficient plants

**Figure 1:**
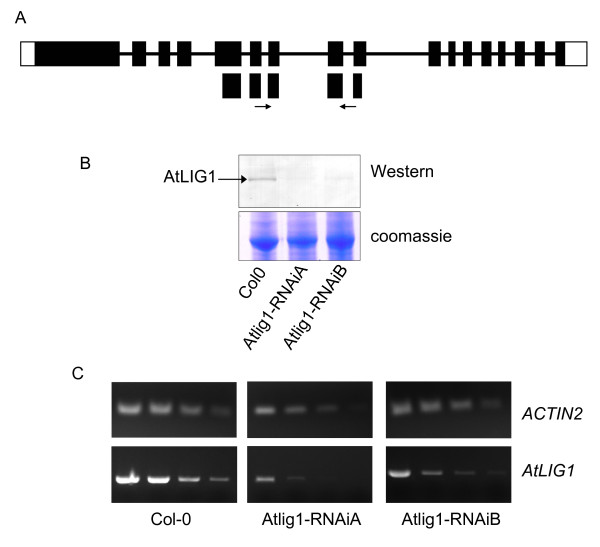
**Silencing DNA ligase I expression in *Arabidopsis thaliana***. a) organisation of the *AtLIG1 *region used for silencing B) Western analysis showing AtLIG1 protein levels in wild type and silenced lines C) RT-PCR analysis of *AtLIG1 *transcript levels in wild type and silenced lines

In the absence of viable knockout lines, *Arabidopsis *plants with reduced levels of LIG1 were generated using an RNAi approach to gain further insight into gene function (Figure 1A). Both *Arabidopsis DNA LIGASE 1 *(*AtLIG1*) transcript and protein levels in the silenced lines were determined by semi-quantitative RT-PCR and Western blotting respectively (Figures 1B and 1C). Two lines with reduced levels of AtLIG1 protein were selected for further analysis and designated *atlig1-RNAiA *and *atlig1-RNAiB*. These plants displayed an approximate four-fold reduction in AtLIG1 protein (Figure 1B), which although resulting in severe growth defects, was sufficient for propagation of these lines through to seed production.

**Figure 2:**
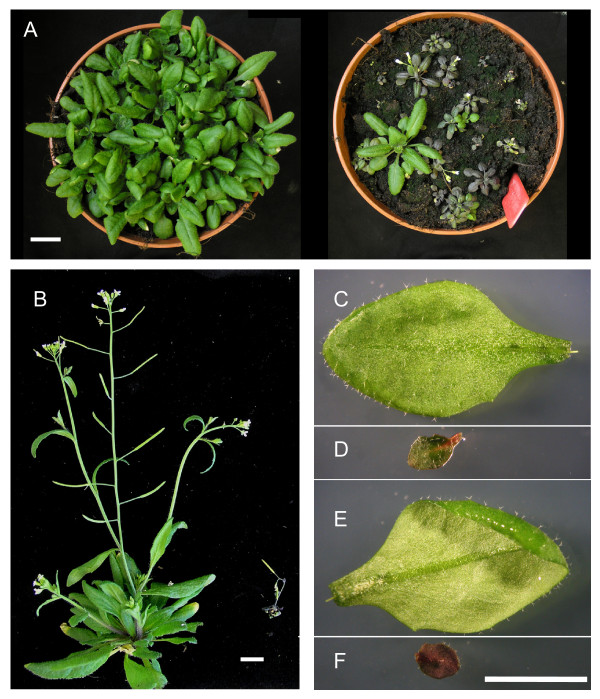
**Phenotypic analyses of AtLIG1 deficient plants**. A) Comparison of wild-type and *atlig1-RNAiA *lines. B) WT and *atlig1-RNAi *plants photographed 6 weeks after germination. Adaxial leaf from WT (C) and *atlig1-RNAi *lines (D) Abaxial surface of WT (E) and *atlig1-RNAi *lines (F). Bar = 1 cm

**Figure 3:**
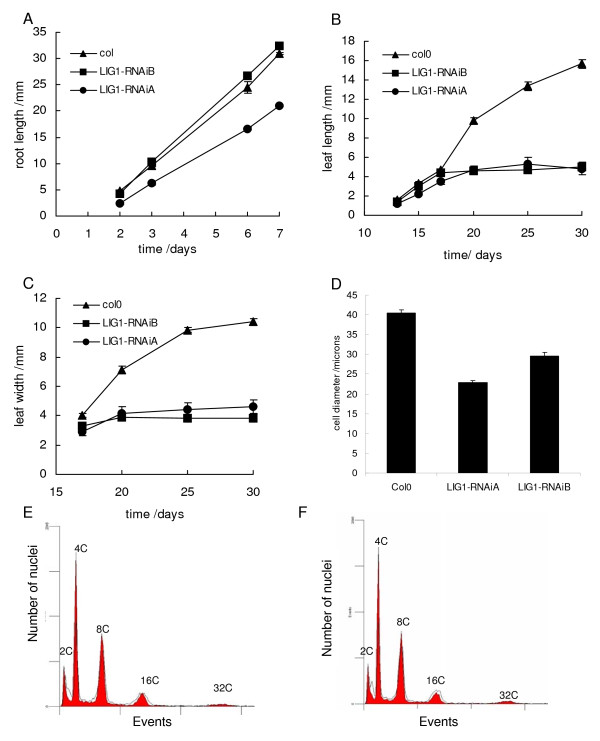
***AtLIG1 *silencing results in reduced tissue and cell size, but endoreduplication is not affected**. A) Root growth in wild-type compared to *atlig1-RNAi *silenced plants B) Leaf length in wild-type compared to *atlig1-RNAi *silenced plants C) Leaf width of the third leaves was measured. D) Protoplast cell size from rosette leaves from plants at first bolting. Error bars indicate SE. E) Flow cytometry of wild type 2 week seedlings with Col-0 (red coloured plot) and *atlig1-RNAiA *(black line). F) Flow cytometry of wild type 2 week seedlings with Col-0 (red coloured plot) and *atlig1-RNAiB *(black line). Error bars indicate SE.

LIG1-deficient plants displayed a stunted and stressed phenotype (Figure 2A–D) which became more pronounced with age. Leaf and root growth were measured to quantify growth differences between AtLIG1-silenced lines and wild type plants. Interestingly the lines with reduced AtLIG1 protein did not display any delay in germination (data not shown). During the first one to two weeks growth roots were significantly smaller in the *atlig1-RNAiA *compared to wild type or *atlig1-RNAiB *plants (p < 0.01 t-Test, Figure 3A–C).

A reduction in length and width of the third and fourth leaves became more pronounced with plant age in both silenced lines relative to wild type controls (Figure 3B, C). By 30 days the average length of the third leaves was 4.2 mm in *atlig1-RNAi *lines as compared to a wild-type value of 15 mm (p < 0.01 t-Test). Corresponding leaf widths were 10.4 mm for the wild-type and significantly less for the RNAi lines at 3.8–4.6 mm (p < 0.01 t-Test). The daily growth rate was 1.25 ± 0.14 mm for wild-type, 0.37 ± 0.10 mm for *atlig1-RNAiA *and 0.35 ± 0.14 mm for *atlig1-RNAiB *line. The final size of mature *Arabidopsis *leaves is a function of both cell division and cell expansion [16]. Therefore, further investigation of the reduced organ size in the *atlig1-RNAi *lines analysed cell size in protoplasts isolated from rosette leaves of wild type and silenced lines after four weeks growth. Cell size was significantly reduced in the *atlig1-RNAi *lines (Figure 3D) with mean cell diameters of 22.9 ± 0.5 μm and 29.6 ± 0.8 μm in the *atlig1-RNAiA *and *atlig1-RNAiB *lines respectively, compared to 40.5 ± 0.8 μm in wild type plants. This 43% and 27% reduction in cell size of *atlig1-RNAiA *and *atlig1-RNAiB *plants respectively was not sufficient to explain the approximate 70% reduction in leaf length and 60% reduction in leaf width observed relative to wild type plants. This indicated that reduced cell number was also responsible for the decreased organ size in the *atlig1-RNAi *lines.

The extent to which cells have undergone endoreduplication is an important factor in the determination of plant cell size [17]. Flow cytometry was performed on the silenced and wild type plants to determine the ploidy levels of leaf cells. Distinct peaks were observed with wild type and the *atlig1-RNAi *lines, corresponding to 2C, 4C, 8C, 16C and 32C, with no significant difference between the wild type and LIG1 depleted lines in terms of peak height (Figure 3E, F). However, the *atlig1-RNAi *lines both displayed an increase in cells between 2C and 4C indicative of slowed progression or arrest in S-phase. This is consistent with a requirement for AtLIG1 not only in DNA replication and may also reflect impairment in DNA repair pathways leading to compromised S-phase. Normal endoreduplication in the *atlig1-RNAi *lines was confirmed by the development of typical tricomes and a wild type-like etiolation response, both of which are compromised in mutants affecting the endocycle [18] (data not shown).

### Analysis of *atlig1-RNAi *single strand break repair kinetics

**Figure 4:**
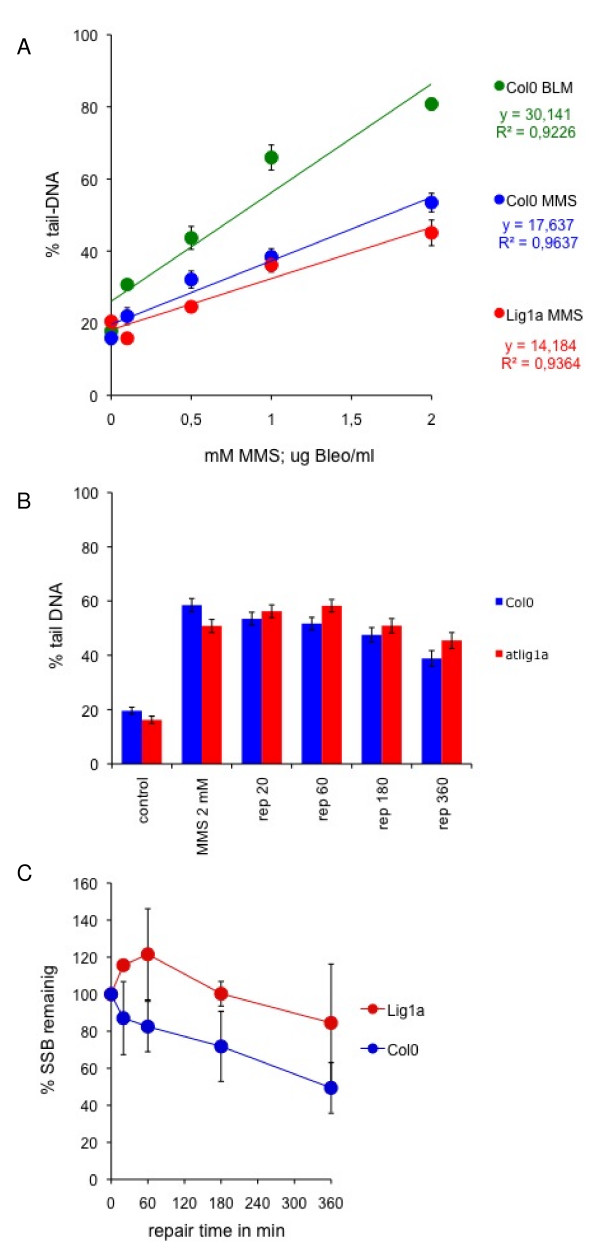
**Kinetics of single strand break repair is altered in the *atlig1-RNAi *lines**. (A) Induction of SSBs by methyl methanesulfonate (MMS). Ten day old seedlings of *Arabidopsis *Col0 were treated with for 1 hour. Nuclei isolated from treated and untreated seedlings were analysed by the alkali/neutral version of comet assay and evaluated for comet formation. The mean percentage of DNA in the comet tail for 300 comets for each concentration of MMS are shown. Induction of SSBs is linear in the 0–2 mM MMS concentrations range (R^2 ^= 0.9638 Col0 and R^2 ^=0.9365 *atlig1-RNAi *respectively). (B) Time course of SSB repair in Col0 and *atlig1-RNAi *lines over 6 hour repair period. Background DNA damage in untreated (control) seedlings and damage after 1 hour treatment with 2mM MMS (t = 0) is similar in both lines. Contrary to wild type plants, the number of SSBs in *atlig1-RNAiA *increases for 60 minutes after the end of treatment suggesting delayed ligation during repair. (C) Kinetics of SSB repair. The percentage of SSBs remaining were calculated for 0, 20, 60, 180 and 360 minute repair time points after the treatment with 2 mM MMS. Maximum damage is normalised as 100% at t = 0 for all lines.

Single cell electrophoresis (Comet) assay under strictly neutral (N/N) or neutral with alkaline unwinding step (A/N) conditions quantifies the repair kinetics of double or single strand DNA breaks respectively [19,20]. The Comet assay was used here to investigate the kinetics of DNA repair in *atlig1-RNAi *lines compared to wild-type plants. DNA single strand breaks were induced by MMS treatment in ten-day old seedlings of wild type and AtLIG1 depleted lines, with a linear dose response curve up to 2 mM MMS (Figure 4A). Background DNA damage contributed around 20% DNA comet tails in untreated (control) seedlings and 60% of comet tail DNA after 1 hour treatment with 2 mM MMS (t = 0). The effects seen were similar in wild type and *atlig1A *lines (Figure 4B). Seedlings treated with 2 mM MMS were analysed using the comet assay and the *atlig1-RNAi *lines displayed reduced repair rates of induced DNA SSB damage in comparison to wild-type with around 50% of damage remaining after 360 min in controls compared to 85% in *atlig1-RNAi *plants (Figure 4C). Notably, *atlig1-RNAi *plants, but not wild type controls, demonstrated an initial increase in SSB accumulation in the first 60 min of recovery following MMS treatment (Figure 4C). This may be attributable to the accumulation of SSBs arising from unrestricted removal of alkylated bases induced by MMS in genomic DNA and a delayed ligation step arising from the limited availability of DNA ligase activity during base excision repair in the RNAi line.

### Reduced rates of DNA double strand break repair in *atlig1-RNAi *lines

**Figure 5:**
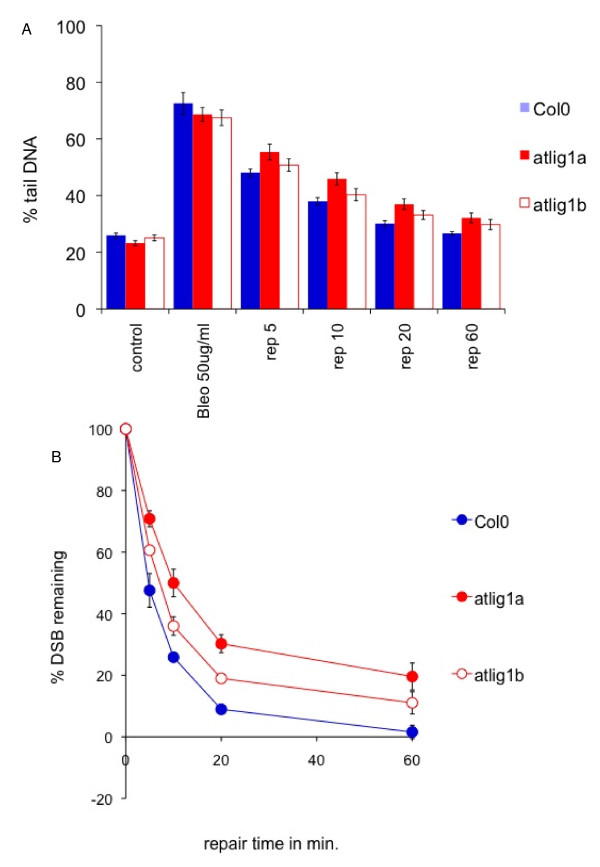
**DSB repair in *Arabidopsis *Col0 and *atlig1-RNAiA *and *atlig1-RNAiB *lines determined by neutral comet assay**. (A) Time course of DSB repair during 1-hour repair period. Background DNA damage in untreated (control) seedlings and damage after 1 hour treatment with 30 μg/ml bleomycin (t = 0) is similar in all lines. Defects in DSB repair is manifested by DNA remaining in comet tails (% tail DNA). (B) Kinetics of DSB repair measured over the first 60 min show biphasic kinetics. Percents of DSB remaining were calculated from % tail DNA as described in Comet data evaluation.

Single cell electrophoresis under neutral conditions was used to analyse the repair of DNA double strand breaks in the wild type and silenced lines. This analysis revealed similar levels of background (non-induced) DNA damage in all mutant and wild-type seedlings, with approximately 25% of DNA migrating in the comet tail (Figure 5A, B). This indicated there was no significant accumulation of DSBs in 10 day old seedlings deficient in AtLIG1 in the absence of genotoxin treatment. As differences in growth between WT and AtLIG1 deficient lines become more pronounced at around 20 days onwards, the effect of diminished levels of AtLIG1 on the long term growth and development of the plants may well be attributable to the accumulation of unrepaired damage.

The radiomimetic bleomycin [21] causes DNA double strand breaks in DNA. A one hour treatment of the ten-day seedlings with the bleomycin (30 μg/ml) resulted in a large shift in the migration of the genomic DNA with 60–80% migrating in the comet tail, indicative of extensive fragmentation, with AtLIG1 deficient and wild type plants displaying similar responses (Figure 5A). Most DSBs were removed within one hour of bleomycin treatment in wild type lines (Figure 5). The kinetics of DSB repair in mutant and wild type plants were then determined by the comet assay over a time course of recovery from bleomycin treatment, with the extent of DNA damage remaining being calculated from the percentage of DNA in the tail (as defined in the Methods). Wild type seedlings displayed very rapid repair of DSBs. The repair was biphasic, with a very rapid initial phase followed by a slower phase in which the small remainder of DNA damage was repaired. The initial rapid removal of the majority of DSBs from genomic DNA followed first order kinetics. Analysis of the first ten minutes following bleomycin treatment found significantly slower DSB repair in the RNAi lines compared to wild type plants with a t 1/2 of 6.7 and 9.1 min for two independent RNAi lines compared to 4.9 min for wild type plants (Figure 5B). These differences led to the presence of a residual 10–20% of DSBs remaining in the RNAi lines at 60 min as compared to hardly detectable levels in wild type plants, equating to the level of DSBs seen in wild type lines at 20 min. This contrasts with the repair kinetics of *atlig4 *mutant plants, which do not display a reduction in the initial rapid repair observed in the *atlig1-RNAi *lines [22]. These results were consistent with a role for AtLIG1 in a novel pathway for the rapid repair of DSBs in plants, although the essential roles of this ligase in plant cells makes it difficult to determine the full extent of the role of AtLIG1 in this pathway.

## Discussion

DNA ligases play essential cellular roles in sealing the phosphodiester backbone during DNA repair and replication. Although a role for *Arabidopsis *LIG4 in NHEJ is well established, the role of the other ligases in *Arabidopsis *DNA repair processes remains unclear. In the present study, the effects of reduced AtLIG1 levels on plant growth and DNA repair kinetics were investigated by analysis of RNAi silenced plant lines.

*AtLIG1 *silenced lines displayed a number of growth defects associated with reduced organ size and activation of stress responses. The slowed leaf growth of AtLIG1 deficient lines as compared to wild-type became increasingly evident with age. This is consistent with a gradual increased accumulation of DNA damage products with leaf age due to reduced levels of AtLIG1 resulting in compromised repair capacity. *AtLIG1 *silenced lines displayed a number of growth defects including reduced organ size and activation of stress responses. The lack of normal AtLIG1 levels resulted in reduced cell size and an increase in cells in S-phase, which over the plant's life span was manifested phenotypically as retarded leaf growth. This becomes increasingly evident with plant age and is consistent with a requirement for AtLIG1 for normal growth and development. The oldest leaves of AtLIG1 deficient plants began to develop a dark green and eventually purple colouration, especially marked on the abaxial leaf surface (Figure 2A–D). The development of this stressed phenotype is similar to previous accounts of the *Arabidopsis *stress response, where the changes in colouration were due to elevated levels of anthocyanin production [2,23]. The oldest leaves eventually bleached, similar to plants exposed to a wide range of treatments including high UVC irradiation [24]. This finding demonstrates that reduction in normal AtLIG1 levels produces phenotypic changes associated with environmental stresses, consistent with the accumulation of DNA damage in the RNAi lines with age. Environmental stresses often induce reactive oxygen species resulting in forms of DNA damage are predominantly repaired via base and nucleotide excision repair pathways. Chronic exposure to these stresses may also result in accumulation of DSBs in the plant genome with time as a consequence of unrepaired single strand breaks being converted into more cytotoxic DSBs [25,26]. The stress response exhibited by the *atlig1-RNAi *lines may be activated by the presence of DNA strand breaks usually associated with oxidative DNA damage. The AtLIG1 deficient plants displayed reduced growth but interestingly the RNAi lines bolted and flowered significantly earlier than wild-type lines (data not shown) in common with previous studies that reported precocious flowering in plants stressed by exposure to low levels of gamma-radiation [25] or UVC [27].

Further analysis investigated the repair kinetics of single and double strand DNA breaks induced in wild type and silenced lines. Of the different forms of DNA damage, DSBs are one of the most cytotoxic and, if left unrepaired, can result in chromosome fragmentation and loss of genetic information. In eukaryotes, DSBs are repaired by homologous recombination or NHEJ pathways. In *Arabidopsis *the NHEJ pathway components KU70, KU80 and LIG4 are all required for survival of gamma irradiated plants [28]. However, several lines of evidence strongly support the existence of end joining pathways which are independent of KU and LIG4 in higher plants. Knockout mutants of classical NHEJ (C-NHEJ) pathway components in higher plants such as *atku80 *and *atlig4 *are able to integrate T-DNA at random sites in the genome with frequencies of between 10–100% of that found in wild type plants [29-31]. Consistent with these observations, illegitimate end-joining is still active in non-homologous end joining mutants, observed by chromosomal fusions and plasmid re-joining assays *in planta *[32,33]. Recent studies revealed that *atlig4 *mutants display rapid rates of DSB repair, similar to those of wild type plants, indicating either that a second ligase activity or an independent pathway can effectively substitute for loss of LIG4 [22]. Analysis of the RNAi lines indicated that AtLIG1 was required for the initial rapid phase of repair, with reduced AtLIG1 levels resulting in an increase in the half life of a DSB. This was not attributable to increased background levels of DSBs in the untreated *atlig1-RNAi *lines, as these basal levels of genome fragmentation were similar to wild type lines. The decreased rates of DSB repair in the silenced plants suggests that AtLIG1 does not simply substitute for AtLIG4 in C-NHEJ, as *atlig4 *mutants do not display a reduction in this initial rapid phase of DSB repair. Rather, these results indicate that AtLIG1 is required for the fast rejoining of the majority of DSBs within 10 min after the removal of bleomycin. While AtLIG4 is not required for the rapid initial phase of DSB repair, *atlig4 *mutants are hypersensitive to genotoxic agents. This suggests that a subset of DSBs may persist in *atlig4 *mutants that cannot be repaired by the rapid, AtLIG1 dependent mechanism. The repair of these DSBs requires the KU and LIG4 mediated slower repair pathway, and failure to eliminate these lesions from the genome results in the IR hypersensitivity of NHEJ mutants. Parallel pathways for end joining have also been identified in mammals, where a LIG4 and KU independent pathway has been characterised [34,35]. The molecular mechanisms of these pathways are beginning to be determined, with one pathway mediated by PARP1 and LIG3 displaying greatest activity in the G2 phase of the cell cycle [35]. *In vitro *studies using human cell extracts showed that both LIG1 and LIG3 can function in microhomology mediated end joining, whereas LIG4 was not required [34]. A significant difference between DSB repair in plants and mammals is the requirement for LIG4 for the rapid repair of DSBs [35] in contrast to the rapid DSB repair observed in *Arabidopsis lig4 *mutant lines [22]. This rapid repair pathway is dependent on the structural maintenance of chromosome (SMC)-like proteins MIM and RAD21.1 and analysis of the RNAi lines suggest a role for LIG1 in this DNA repair pathway. Future studies will further delineate the molecular mechanism of this repair pathway in plants.

## Conclusion

While *atlig1 *null mutants are non-viable, plants with reduced AtLIG1 levels display growth defects, reduced cell size and a greater proportion of cells in S-phase, consistent with roles for *Arabidopsis *DNA ligase 1 in both DNA repair and DNA replication pathways. Additionally *atlig1-RNAi *plants show reduced rates of DNA repair, including a significant delay in the initial rapid phase of DSB repair. These results indicate that AtLIG1 is required for the rapid KU/LIG4 independent repair of DSBs in plants.

## Methods

### Generation and characterisation of AtLIG1 – RNAi silenced lines

Vector pFGC5941 (TAIR) was used for generation of the silencing constructs [36]. This has a CaMV 35S promoter to drive the expression of the inverted repeat target sequence separated by a 1,352-bp ChsA intron from the petunia Chalcone synthase A gene to stabilize the inverted repeat of the target gene fragment. A 458 bp region of AtLIG1 was amplified by PCR with primers incorporating XbaI and SwaI sites for the forward primer: 5'-GGTCTAGAGGCGCGCCGATACTGAATAAATTCCAGGACATC-3' (LIG1if) and AscI and BamHI sites for the reverse primer: 5'-GGTGGGATCCATTTAAATCATCGATATCGTTAGATGTTACAG-3' (LIG1ir). The PCR product was cloned into pFGC5941 in a two-step cloning procedure that integrates the fragment in opposite orientations on either side of the ChsA intron. The RNAi construct was then used to transform *Arabidopsis *allowing plant selection by basta resistance. The extent of AtLIG1 silencing in plants was determined by Western analysis of AtLIG1 protein levels (Fig 1A). Polyclonal antiserum was raised to full length AtLIG1 overexpressed in *E. coli*. AtLIG1 cDNA [36] was cloned into the plasmid pCal-c (Stratagene) and expressed with a C-terminal calmodulin binding protein (CBP) tag. Expression was induced by the addition of isopropylthiogalactoside (1 mM) for 3 h in *E. coli *strain BL21 (DE3) pLysS (Promega). Bacteria were recovered by centrifugation, resuspended in RS buffer (50 mM Tris-Cl pH 7.5, 50 mM NaCl, 2 mM CaCl_2_, 5% (v/v) glycerol, 0.1% (v/v) Triton X100) and lysed by freeze thawing and sonication. The extract was cleared by centrifugation at 13 000 g for 10 min, applied to a calmodulin affinity resin (Stratagene) and washed with RS buffer. Purified AtLIG1 protein was eluted in 50 mM Tris-Cl pH 7.5, 50 mM NaCl, 2 mM EGTA, 5% (v/v) glycerol, 0.1% (v/v) Triton X100. Further purification was achieved by preparative SDS-PAGE and coomassie stained bands were electroeluted (BioRad) and used for immunisation. In Western analysis of *Arabidopsis *cell extracts, antiserum to AtLIG1 (but not preimmune) identified a band of the expected molecular weight, detected using alkaline phosphatase coupled anti-sheep IgG secondary antiserum and visualised by incubation with nitrotetrazolium blue chloride/5-bromo-4-chloro-3-indolyl phosphate (Sigma).

### Comet assay

DSBs were detected by a neutral comet assay [37] and SSB by A/N version of comet assay as described previously [20,38] In brief, approximately 100 mg of frozen tissue was cut with a razor blade in 500 μl PBS+10 mM EDTA on ice and tissue debris removed by filtration through 50 μm mesh funnels (Partec, Germany) into Eppendorf tubes on ice. 30 – 50 μl of nuclei suspension was dispersed in 300 μl of melted 0.5% agarose (GibcoBRL, Gaithersburg, USA) at 40°C. Four 80 μl aliquots were immediately pipetted onto each of two coated microscope slides (in duplicates per slide) on a 40°C heat block, covered with a 22 × 22 mm cover slip and then chilled on ice for 1 min to solidify the agarose. After removal of cover slips, slides were dipped in lysing solution (2.5 M NaCl, 10 mM Tris-HCl, 0.1 M EDTA, 1% N-lauroyl sarcosinate, pH 10) on ice for at least 1 hour to dissolve cellular membranes and remove attached proteins. The whole procedure from chopping tissue to dipping into lysing solution takes approximately 3 minutes. After lysis, slides were twice equilibrated for 5 minutes in TBE electrophoresis buffer to remove salts and detergents and then electrophoresed at 1 V/cm (app. 20 mA) for 5 minutes. After electrophoresis, slides were dipped for 5 min in 70% EtOH, 5 min in 96% EtOH and air-dried. Comets were viewed in epifluorescence Nikon Eclipse 800 microscope after staining with GelRed stain (Biotium, Hayward, USA) and evaluated by Comet module of Lucia cytogenetics software suite (LIM, Praha, Czech Republic). Wild-type Col0 were used as controls for experiments with *atlig1-RNAiA *and *atlig1-RNAiB*.

### Comet data evaluation

The comet slides were coded and blind evaluated. The fraction of DNA in comet tails (% tail-DNA) was used as a measure of DNA damage. Data in this study were measured in at least 3 independent experiments, each starting with newly grown *Arabidopsis *seedlings. In each experiment, the % tail-DNA was measured at 5 time points: 0, 20, 60, 180 and 360 minutes after treatment and in control seedlings without treatment. Measurements included 4 independent gel replicas of 25 evaluated comets totalling at least 300 comets analyzed per experimental point [38]. The percentage of damage remaining as plotted in Figure 4 and 5 after a given repair time (t_x_) is defined as:



### Preparation of polyclonal antiserum to *Arabidopsis *ligase 1 protein

The ORF of the cloned *Arabidopsis thaliana *ligase 1 cDNA in the pCR2.1 vector (Invitrogen, Leek, The Netherlands) was amplified with primers containing BglII (AGCAAGATCTTTAAACAATAGTTATCTTGGGATCA) and NheI (TGCAACATATGGCGTCGACAGTCTCAG) sites at the 5' and 3' ends of the ORF respectively using the proof-reading DNA polymerase *Pfu *(Stratagene, UK). The DNA fragment was introduced at the NdeI site of the pET-11b vector (Calbiochem-Novabiochem, Nottingham, UK) in frame with a 6×His tag coding region, yielding 6×His-ligase 1 *E. coli *BL21(DE3)pLysS was transformed with 6×His *-PHR1 *and cultured in LB medium supplemented with 50 mg.l^-1 ^ampicilin until an OD 600 nm of 0.6–1.0 was reached. Expression of 6×His-ligase 1 was induced by the addition of 1 mM isothiopropylgalactoside and growth continued for 3 h. Bacterial cell extracts were prepared by sonication of 36 ml cells suspended in 50 mM Tris-HCl, pH 7.5, 2 mM EDTA, 0.1% Triton X-100 (1:10 buffer: culture media ratio used) and incubated with 100 μg.ml^-1 ^lysozyme at 30°C for 15 min. The resultant suspension was centrifuged at 10 000 *g *for 30 min at 4°C. The pelleted proteins were purified by preparative SDS-PAGE using 10% acrylamide gels. The 6×His-AtLIG1 band was excised from the Coomassie-blue stained gel and destained using several changes of 100 mM Tris-HCl pH 7.0. Protein was electroeluted from the gel slices, mixed with Freud's adjuvent and used to raise antiserum in sheep (Scottish Antibody Production Unit, Carluke, Scotland).

### Western blotting

Proteins were extracted from wheat tissue as described by Pang and Hays (1991). Protein concentrations were determined by the Bio-Rad protein assay (Bio-Rad Laboratories, Hemel Hempstead, UK) using bovine serum albumin (BSA) as a standard. Protein samples were separated by SDS-PAGE (10% gel) and transferred to PVDF membrane (Bio-Rad) for 3 h at 100 V. The blots were probed with anti-ligase 1 antiserum. The immune complexes were detected by alkaline-phosphatase conjugated antisheep IgG (Sigma-Aldrich, Poole, UK) and developed using premixed BCIP/NBT solution (Sigma). Primary and secondary antisera were used at 1/10000 and 1/30000 dilutions respectively.

### Flow cytometry

Ploidy analysis was performed using the Cystain absolute P kit (Partec) according to the manufacturer's instructions. Plant nuclei were isolated by chopping fresh leaf material in extraction buffer (Partec) before filtering through a 50 μm membrane. RNase digestion and staining with propidium iodide was followed by analysis using a Becton Dickinson FACSCalibur cytometer.

### Cell size

Cell size was determined by brightfield microscopy (Zeiss LSM 510 META Axiovert 200 M inverted confocal microscope) and image analysis using LSM Image browser software (Zeiss).

## Abbreviations

NHEJ: non-homologous end joining; SSB: Single strand break; DSB: Double strand break; AtLIG1: *Arabidopsis *DNA ligase I; MMS: Methyl methanesulfonate

## Authors' contributions

WMW, JK, CMP and CEW performed the experiments. CEW, CMB and KJA designed the experiments and WMW, KJA, CMB and CEW wrote the manuscript. All authors read and approved the final version of the manuscript.
